# ARNTL2 upregulation of ACOT7 promotes NSCLC cell proliferation through inhibition of apoptosis and ferroptosis

**DOI:** 10.1186/s12860-022-00450-5

**Published:** 2023-03-31

**Authors:** Tao Wang, Kai Wang, Xu Zhu, Nan Chen

**Affiliations:** 1 Affiliated Hospital of Guizhou Medical University, Guiyang, China; 2 The Third Affiliated Hospital of Kunming Medical University, Yunnan Cancer Hospital, Kunming, China

**Keywords:** NSCLC, ARNTL2, ACOT7

## Abstract

**Background:**

Recent studies have reported that the circadian transcription factor aryl hydrocarbon receptor nuclear translocator like 2 (ARNTL2) promotes the metastatic progression of lung adenocarcinoma. However, the molecular mechanisms of ARNTL2 in non-small cell lung cancer (NSCLC) cell growth and proliferation remain to be explored.

**Methods:**

The expression of ARNTL2 and acyl-CoA thioesterase 7 (ACOT7) in lung cancer patients was analyzed based on TCGA database. Gain-of-function of ARNTL2 and ACOT7 was conducted by transfecting the cells with plasmids or lentivirus. Knockdown assay was carried out by siRNAs. Western blot and qRT-PCR were performed to check the protein and mRNA expression. Dual luciferase and ChIP-qPCR assay was applied to check the interaction of ARNTL2 on ACOT7’s promoter sequence. Triglyceride level, MDA production, the activity of casapase 3 to caspase 7, and lipid ROS were measured by indicated assay kit. Cellular function was detected by CCK8, colony formation and flow cytometry analysis of cell death and cell cycle.

**Results:**

We demonstrated that ARNTL2 upregulation of ACOT7 was critical for NSCLC cell growth and proliferation. Firstly, overexpression of ARNTL2 conferred the poor prognosis of LUAD patients and supported the proliferation of NSCLC cells. Based on molecular experiments, we showed that ARNTL2 potentiated the transcription activity of ACOT7 gene via direct binding to ACOT7’s promoter sequence. ACOT7 high expression was correlated with the worse prognosis of LUAD patients. Gain-of-function and loss-of-function experiments revealed that AOCT7 contributed to NSCLC cell growth and proliferation. ACOT7 regulated the apoptosis and ferroptosis of NSCLC cells, while exhibited no effect on cell cycle progression. ACOT7 overexpression also potentiated fatty acid synthesis and suppressed lipid peroxidation. Lastly, we showed that ARNTL2 knockdown and overexpression inhibited and promoted the cellular triglyceride production and subsequent cell proliferation, which could be reversed by ACOT7 overexpression and knockdown.

**Conclusion:**

Our study illustrated the oncogenic function of ARNTL2/ACOT7 axis in the development of NSCLC. Targeting ARNTL2 or ACOT7 might be promising therapeutic strategies for NSCLC patients with highly expressed ARNTL2.

**Supplementary Information:**

The online version contains supplementary material available at 10.1186/s12860-022-00450-5.

## Introduction

Lung cancer is the leading cause of cancer-related death among all the malignancies worldwide [1]. With approximately 2 million new cases diagnosed, lung cancers are histologically classified into two subtypes, including small cell lung carcinoma (SCLC) and non-small cell lung cancer (NSCLC), the latter accounting for 85% of the cases [2]. Benefitting from the intensive genomics, transcriptomics and proteomics studies, promising therapeutic targets and effective drugs have been developed against to this deadly malignancy [3]. Although the targeted therapy, including EGFR inhibitors [4], or the immunotherapy, such as PD-1 antibodies [5], have gain prominent success on the treatment of lung cancers, only some patients show durable response to these drugs. Identifying novel drivers may help us develop specific therapeutic options which can benefit for the remaining patients.

Recent studies have shown that dysregulation of circadian clock plays an essential role during the progression of various cancers [6]. The most well-known genes regulating circadian rhythms include Clock, Period 1, Period 2, Period 3, casein kinase Iε (CKIε), cryptochrome 1 (Cry1), chryptochrome 2 (Cry2), Bmal1, as well as its paralog Bmal2 (ARNTL2). Comparing with other core circadian rhythms genes, the relevance of ARNTL2 in carcinogenesis is less reported. Some evidences have shown that ARNTL2 acts as oncogene in cancers. For instance, upregulation of ARNTL2 is a worse prognosis factor for clear cell renal cell carcinoma [7]. Depletion of ARNTL2 suppresses the malignant growth of colorectal carcinoma through inhibiting the expression of SMOC2 and the activity of PI3K/AKT signaling pathway [8]. In addition, oncogenic function of ARNTL2 is reported in other cancer types, including pancreatic ductal adenocarcinoma [9], breast cancer [10], as well as lung cancer [11]. Based on these studies, it seems that ARNTL2 is pivotal for the metastasis and invasion of malignant tumors. However, more studies should be conducted to explore the role and underlying mechnisms of ARNTL2 in the growth and proliferation of cancer cells, such as lung cancer.

In this study, we investigated the function of ARNTL2 and its potential mechanism in NSCLC by analyzing TCGA public database, performing gain-of-function and loss-of-function experiments, as well as dual luciferase reporter and ChIP-qPCR assay. Our results may provide novel evidences on the role and molecular mechanisms of ARNTL2 in NSCLC.

## Results

### Overexpression of ARNTL2 contributes to the progression of NSCLC patients and the proliferation of NSCLC cells

**Fig. 1:**
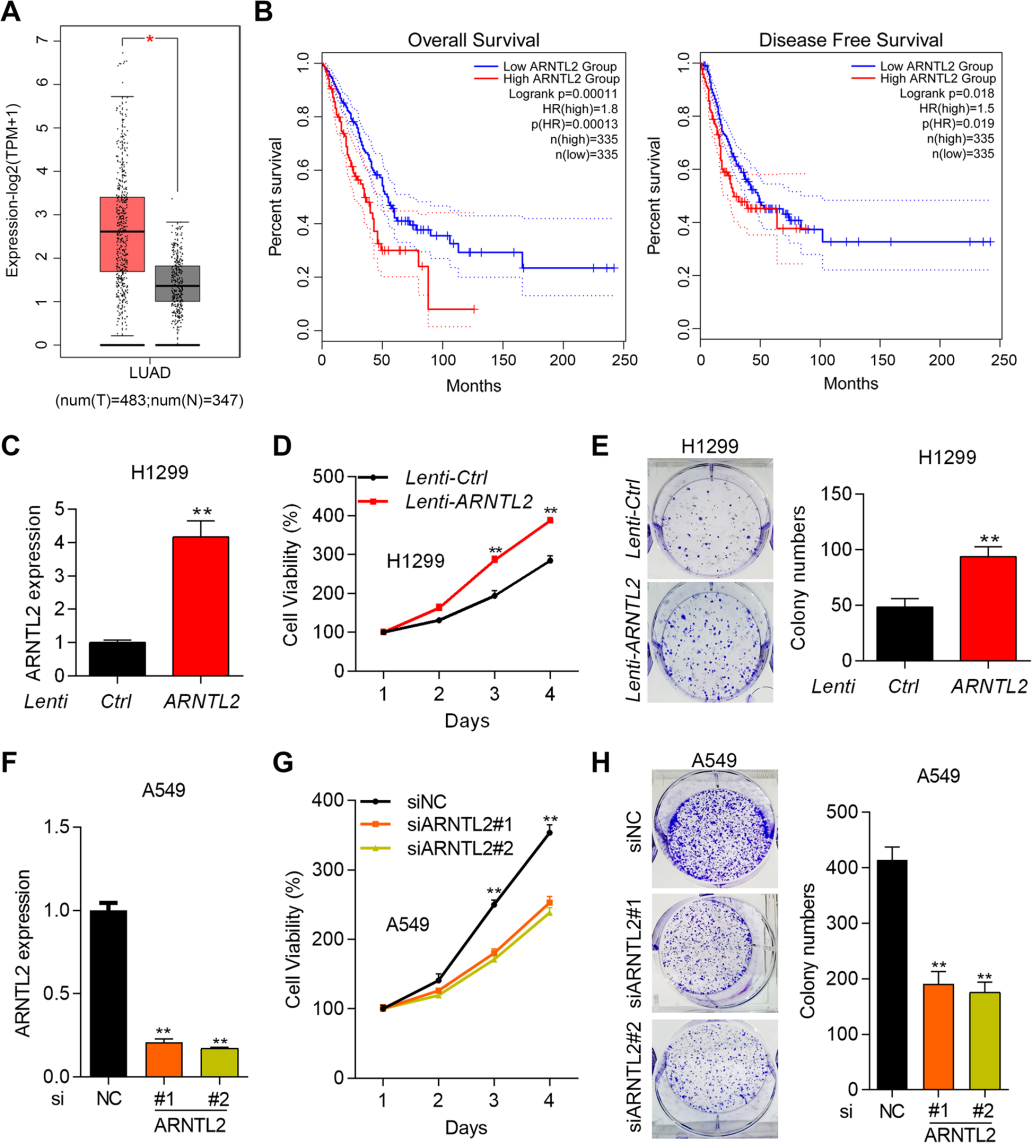
Overexpression of ARNTL2 contributes to the progression of NSCLC patients and the proliferation of NSCLC cells. **A** The mRNA expression of ARNTL2 was analyzed in LUAD (*n* = 483) and normal (*n* = 347) tissues based on the TCGA database *p* < 0.05. **B** Overall and disease-free survival was analyzed for LUAD patients who were divided into ARNTL2 high and low expression group. *n* = 335 per group. *p* = 0.00011 and *p* = 0.018. **C** RT-qPCR analysis of ARNTL2 in H1299 cells transfected with Ctrl and ARNTL2 overexpressing lentivirus. **p < 0.01. **D** and **E** Cell proliferation was determined by CCK8 (**D**) and colony formation (**E**) assay in H1299 cells transfected with Ctrl and ARNTL2 overexpressing lentivirus. **p < 0.01. **F** RT-qPCR analysis of ARNTL2 in A549 cells transfected with siNC, siARNTL2#1 and siARNTL2#2. **p < 0.01. **G** and **H** Cell proliferation was determined by CCK8 (**G**) and colony formation (**H**) assay in A549 cells transfected with siNC, siARNTL2#1 and siARNTL2#2. **p < 0.01

TCGA is a public database which could be applied to analyze the clinical relevance of various genes in cancer patients. We firstly used TCGA to examine the expression of ARNTL2 in lung adenocarcinoma (LUAD) and lung squamous cell carcinoma (LUSC) patients. The results showed that ARNTL2 was significantly upregulated in LUAD and LUSC tissues compared with normal tissues (Fig. 1A and supplementary Fig. 1A, *p* < 0.05). We further analyzed the correlation between ARNTL2’s expression and the patients’ survival. The patients were divided into ARNTL2 high and low expression group. ARNTL2 high expression predicted shorter overall (*p* = 0.00011) and disease free (*p* = 0.019) survival compared with ARNTL2 low expression in LUAD patients (Fig. 1B), suggesting that ARNTL2 is a worse prognosis factor for LUAD patients. However, ARNTL2 expression was not significantly correlated with the overall (*p* = 0.67) and disease free (*p* = 0.25) survival of LUSC patients (Supplementary Fig. 1B). To illustrate the effect of ARNTL2 on proliferation, we overexpressed and knocked down ARNTL2 in NSCLC cells. Firstly, we checked the expression of ARNTL2 in normal lung cells Beas-2B and in three NSCLC cell lines, including A549, H1299 and H1975. The results showed that ARNTL2 expression was higher in A549, H1299 and H1975 cells as compared with Beas-2B cells (Supplementary Fig. 2). Among the NSCLC cells, ARNTL2 was highest in A549 cells and was relatively lower in H1299 cells (Supplementary Fig. 2). Therefore, we overexpressed ARNTL2 in H1299 cells and knocked down ARNTL2 in A549 cells to study the role of ARNTL2 in NSCLC. Overexpression of ARNTL2 by lentivirus dramatically accelerated the proliferation and colony formation of H1299 cells (Fig. 1C-1E, *p* < 0.01). By contrast, knockdown of ARNTL2 by two siRNAs significantly suppressed the viability of A549 cells (Fig. 1F-1H, *p* < 0.01). These results suggest that ARNTL2 functions as a worse prognosis biomarker and an oncogenic protein in NSCLC.

### ARNTL2 potentiates the transcription activity of ACOT7 via direct interaction on its promoter sequence

**Fig. 2:**
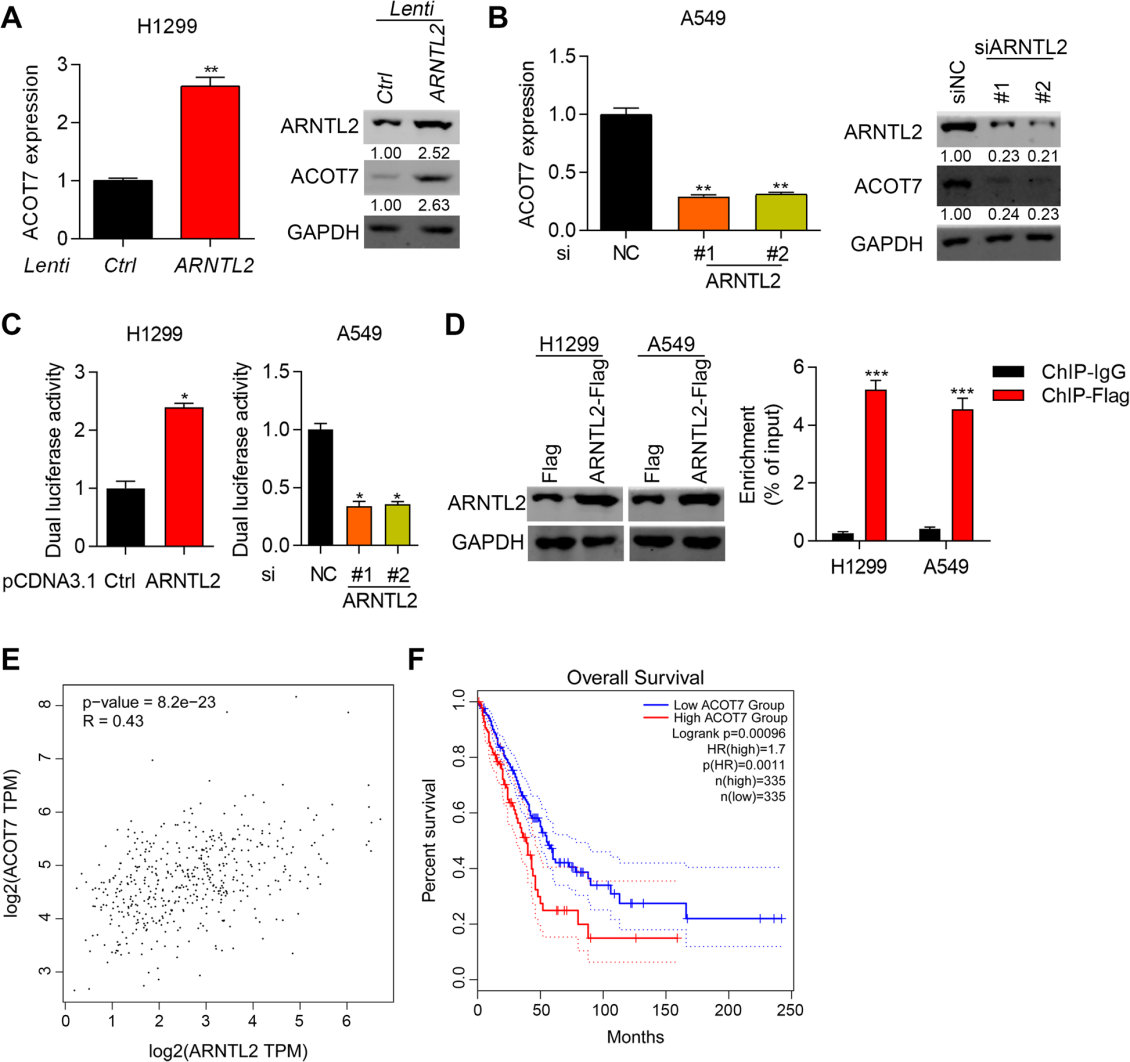
ARNTL2 potentiates the transcription activity of ACOT7 via direct interaction to its promoter sequence. **A** RT-qPCR and immunoblotting analysis of ARNTL2 in H1299 cells transfected with Ctrl and ACOT7 overexpressing lentivirus. Quantification of immunoblotting results was presented. **p < 0.01. **B** RT-qPCR and immunoblotting analysis of ACOT7 in A549 cells transfected with siNC, siARNTL2#1 and siARNTL2#2. Quantification of immunoblotting results was presented. *p < 0.05. **C** Dual luciferase activity of ACOT7 promoter was assessed in H1299 cells with ARNTL2 overexpressiong and in A549 cells with ARNTL2 knockdown. *p < 0.05. **D** Immunoblotting analysis of ARNTL2 in H1299 and A549 cells transfected with pCDNA3.1-Flag (Flag) and pCDNA3.1-ARNTL2-Flag (ARNTL2-Flag). Chip-qPCR was performed to determine the binding of ARNTL2 to the promoter sequence of ACOT7. ***p < 0.001. **E** Sperman correlation between ARNTL2 and ACOT7 in LUAD patients based on TCGA database. *p* < 0.0001. **F** Overall survival was analyzed for LUAD patients who were divided into ACOT7 high and low expression group. *n* = 335 per group. *p* = 0.00096. The blots were cut prior to hybridisation with antibodies during immunoblotting experiments

Since ARNTL2 is a transcription factor, there must be downstream effectors which are responsible for the tumor promoting function of ARNTL2 in NSCLC. Circadian has been shown to play an essential role in regulating cellular metabolism [12]. As the central regulator of circadian, whether ARNTL2 regulates cellular metabolism, such as lipid metabolism, should be investigated. We focused on acyl-CoA thioesterase (ACOT) family because the members of this family exhibited important role in regulating fatty acid production. Based on RT-qPCR and immunoblotting results, we showed that ARNTL2 overexpression promoted the mRNA and protein expression of ACOT7 (Fig. 2A, *p* < 0.01). Inverse results were found in A549 cells after knocking down ARNTL2 (Fig. 2B, *p* < 0.05). Dual luciferase reporter assay demonstrated that the luciferase activity of ACOT7 promoter was increased and decreased in NSCLC cells after ectopically expressing and downregulating ARNTL2, respectively (Fig. 2C, *p* < 0.05). To explore the interaction between ARNTL2 and the promoter sequence of ACOT7 in H1299 and A549 cells, we transfected the cells with pCDNA3.1-ARNTL2-Flag vectors and subjected the cells to Chip-qPCR experiments. Western blot results showed that ARNTL2 was overexpressed in H1299 and A549 cells transfected with pCDNA3.1-ARNTL2-Flag vectors (Fig. 2D). Chip-qPCR results showed that ARNTL2 directly bound to the promoter sequence of ACOT7 (Fig. 2D, *p* < 0.001). These results suggest that ARNTL2 positively regulates the expression of ACOT7 through direct interaction on its promoter. We further analyzed the relationship between ARNTL2 and ACOT7, as well as the prognosis value of ACOT7 in LUAD patients. The results showed that ARNTL2 was positively correlated with ACOT7 in LUAD patients (Fig. 2E, *p* < 0.0001). In addition, ACOT7 high expression predicted poor prognosis of LUAD patients (Fig. 2F, *p* = 0.00096). Taken together, ARNTL2 upregulation of ACOT7 confers worse prognosis of LUAD patients.

### ACOT7 promotes the growth and proliferation of NSCLC cells

**Fig. 3:**
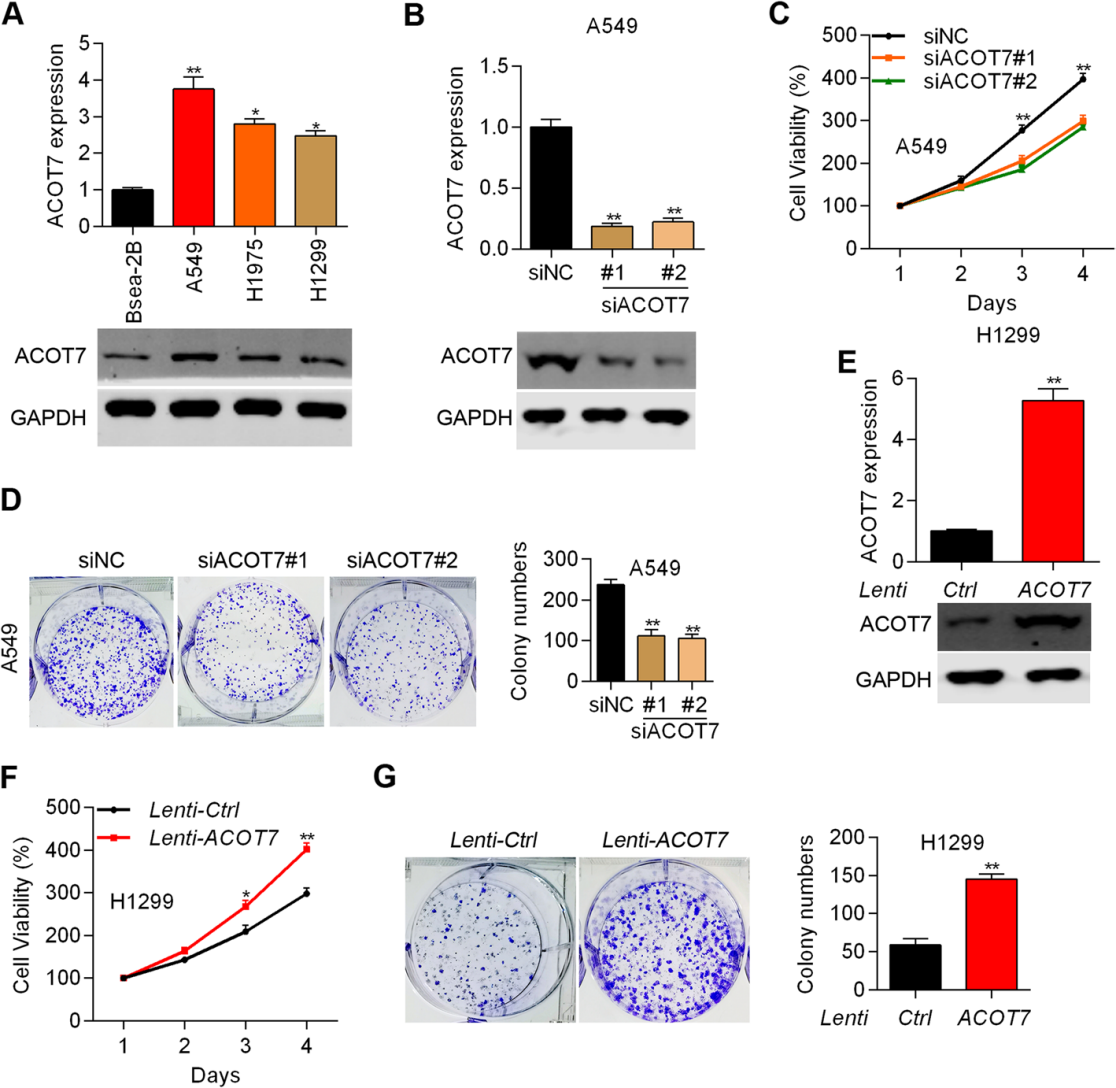
ACOT7 promotes the growth and proliferation of NSCLC cells. **A** RT-qPCR and immunoblotting analysis of ACOT7 in Beas-2B, A549, H1975 and H1299 cells. *p < 0.05. **p < 0.01. **B** RT-qPCR and immunoblotting analysis of ACOT7 in siNC, siACOT7#1 and siACOT7#2 A549 cells. **p < 0.01. **C** and **D** Cell proliferation was tested by CCK8 (**C**) and colony formation (**D**) assay in siNC, siACOT7#1 and siACOT7#2 A549 cells. **p < 0.01. **E** RT-qPCR and immunoblotting analysis of ACOT7 in H1299 cells transfected with Ctrl and ACOT7 overexpression lentivirus. **p < 0.01. **F** and **G** Cell proliferation was tested by CCK8 (**F**) and colony formation (**G**) assay in H1299 cells transfected with Ctrl and ACOT7 overexpression lentivirus. *p < 0.05. **p < 0.01. The blots were cut prior to hybridisation with antibodies during immunoblotting experiments

To study the role of ACOT7 in NSCLC cell growth, we checked the expression of ACOT7 in normal lung cells Beas-2B and in NSCLC cells, including A549, H1975 and H1299. RT-qPCR and immunoblotting results showed that ACOT7 expression was higher in the NSCLC cells compared with Beas-2B cells (Fig. 3A). Because ACOT7 was highest in A549 cells, we knocked down ACOT7 in A549 cells by transfecting the cells with siRNAs (Fig. 3B). We found that knockdown of ACOT7 obviously suppressed the proliferation and colony formation of A549 cells (Fig. 3C and 3D). By contrast, we overexpressed ACOT7 in H1299 cells (Fig. 3E). As expected, ectopic expression of ACOT7 promoted the proliferation and growth of H1299 cells (Fig. 3F and G). Collectively, ACOT7 acts as an oncogenic protein in NSCLC cells.

Next, we analyzed whether ACOT7 regulated cell cycle by staining the cells with PI and analyzed on flow cytometry. We showed that ACOT7 had no significant effect on the cell cycle progression of NSCLC cells (Supplementary Fig. 3).

### ACOT7 suppresses apoptosis and ferroptosis

**Fig. 4:**
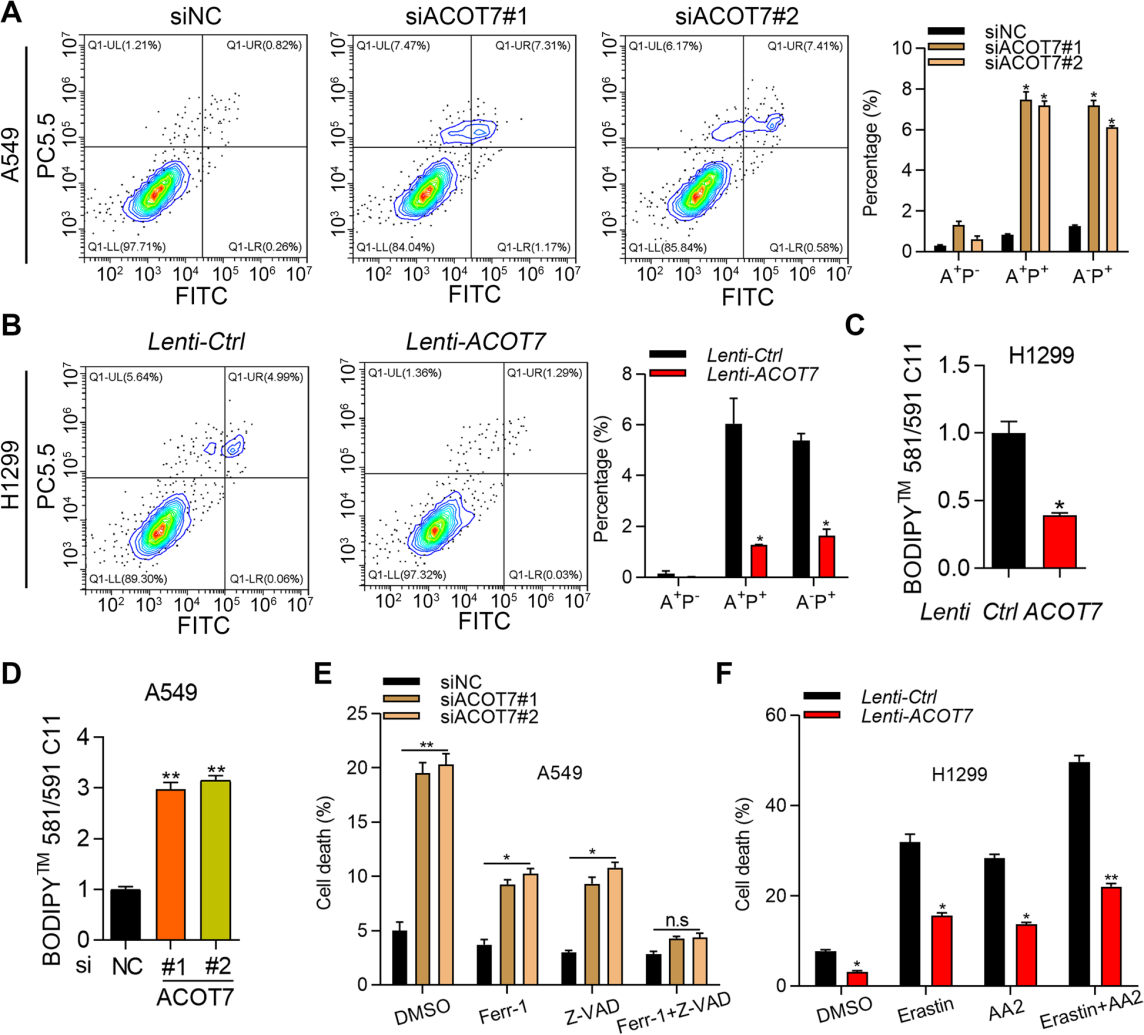
ACOT7 suppresses apoptosis and ferroptosis. **A** siNC, siACOT7#1 and siACOT7#2 A549 cells were stained with PI and Annexin V and then subjected to flow cytometry analysis. A^+^P^−^, Annexin V^+^PI^−^; A^+^P^+^, Annexin V^+^PI^+^; A^−^P^+^, Annexin V^−^PI^+^. *p < 0.05. **B** H1299 cells transfected with Ctrl and ACOT7 overexpression lentivirus were stained with PI and Annexin V and then subjected to flow cytometry analysis. A^+^P^−^, Annexin V^+^PI^−^; A^+^P^+^, Annexin V^+^PI^+^; A^−^P^+^, Annexin V^−^PI^+^. *p < 0.05. **C** and **D** Lipid peroxidation was examined by using BODIPY 581/591C11 in H1299 cells transfected with *Lenti-Ctrl* and *Lenti-ACOT7*, and in A549 cells transfected with siNC, siACOT7#1 and siACOT7#2. *p < 0.05. **p < 0.01. **E** siNC, siACOT7#1 and siACOT7#2 A549 cells were treated with DMSO, ferrostatin-1 (Ferr-1, 2 uM), Z-VAD-FMK (Z-VAD, 8 ug/ml) and Ferr-1 + Z-VAD and cell viability was tested by trypan blue staining. *p < 0.05. **p < 0.01. n.s., not significant. **F** H1299 cells transfected with Ctrl and ACOT7 overexpression lentivirus were treated with DMSO, erastin (5 uM), apoptosis activator 2 (AA2, 4 uM) and erastin + AA2 and cell viability was tested by trypan blue staining. *p < 0.05. **p < 0.01

Next, we performed PI/Annexin V staining to examine which cell death type was regulated by ACOT7. As shown by flow cytometry analysis, we found that ACOT7 downregulation dramatically enhanced the percentage of Annexin V^+^PI^+^ cells (Fig. 4A). On the contrary, ACOT7 overexpression suppressed the percentage of Annexin V^+^PI^+^ cells (Fig. 4B). In addition, the activity of caspase 3 to caspase 7 was negatively regulated by ACOT7 (Supplementary Fig. 4). These results suggest that ACOT7 suppresses the apoptosis of NSCLC cells. Since the Annexin V^−^PI^+^ cells were also regulated by ACOT7 knockdown and overexpression (Fig. 4A and B), we predicted that ACOT7 might influence other cell death type, such as ferroptosis. To dissect this question, we stained the cells with C-11 BODIPY and subjected them to lipid ROS detection on flow cytometer. The results showed that ACOT7 overexpression suppressed, while ACOT7 knockdown enhanced the lipid ROS in NSCLC cells (Fig. 4C and D). Furthermore, we treated siNC, siACOT7#1 and siACOT7#2 A549 cells with ferroptosis inhibitor Ferr-1, apoptosis inhibitor Z-VAD or their combination. We showed that either Ferr-1 or Z-VAD partly reversed the cell death induced by ACOT7 knockdown, and their combination could completely reverse the phenotype (Fig. 4E). Then we treated Ctrl and ACOT7 overexpressing H1299 cells with ferroptosis inducer erastin, apoptosis inducer AA2 or their combination. The results showed that ACOT7 overexpression significantly rescued the cell death induced by erastin, AA2 and their combination (Fig. 4F). Taken together, ACOT7 suppresses both apoptosis and ferroptosis in NSCLC cells.

### ACOT7 promotes the production of monounsaturated fatty acids and lipid peroxidation

**Fig. 5:**
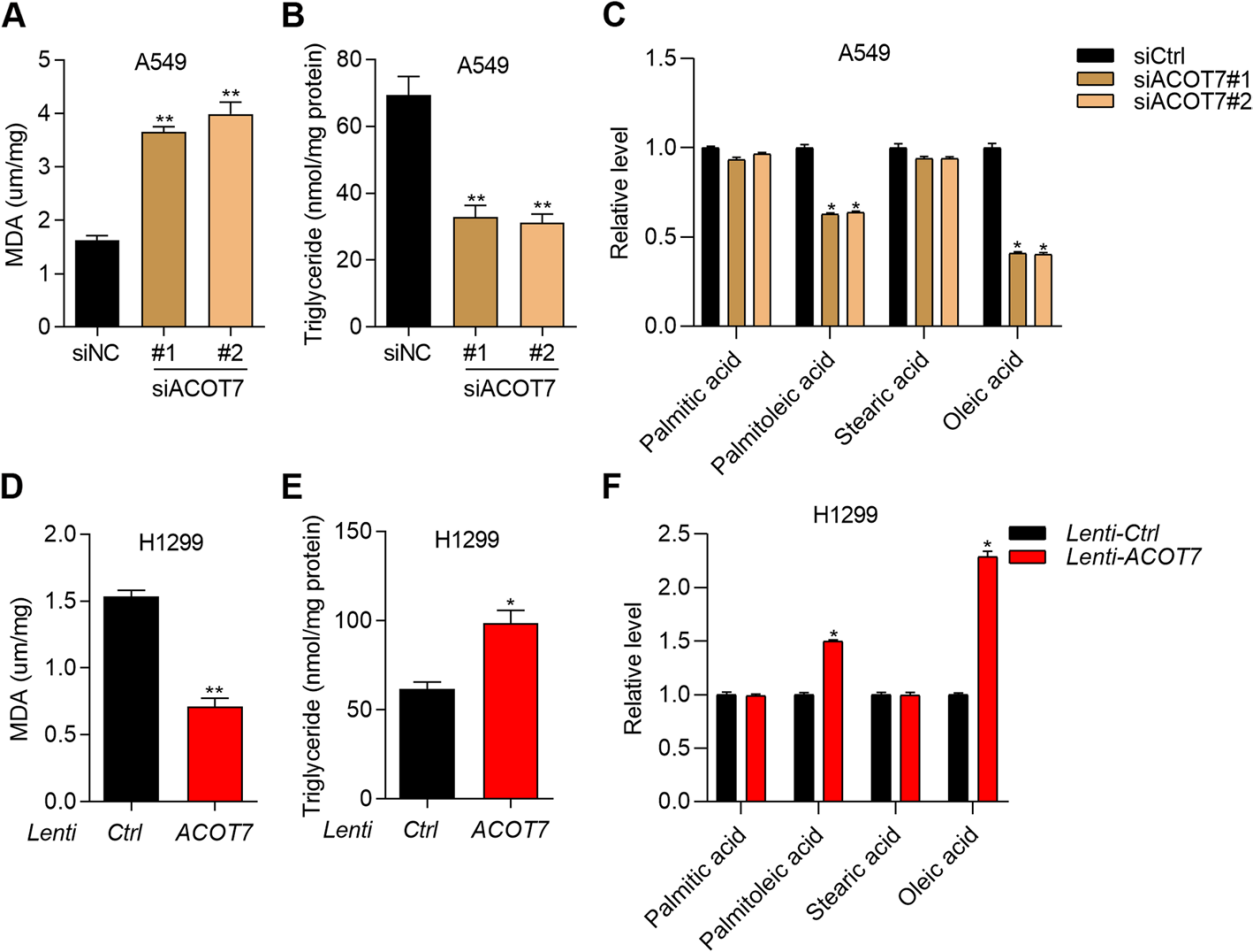
ACOT7 promotes the production of oleic acid and lipid peroxidation. **A** and **B** MDA (**A**) and triglyceride (**B**) abundance was tested by indicated assay kit in siNC, siACOT7#1 and siACOT7#2 A549 cells. **p < 0.01. **C** The level of palmitic acid, palmitoleic acid, stearic acid, and oleic acid was measured in siNC, siACOT7#1 and siACOT7#2 A549 cells. *p < 0.05. **D** and **E** MDA (**D**) and triglyceride (**E**) abundance was tested by indicated assay kit in H1299 cells transfected with Ctrl and ACOT7 overexpression lentivirus. *p < 0.05. **p < 0.01. **F** The level of palmitic acid, palmitoleic acid, stearic acid, and oleic acid was measured in H1299 cells transfected with Ctrl and ACOT7 overexpression lentivirus. *p < 0.05

ACOT7 is an important regulator of fatty acid metabolism. Dysregulated fatty acid synthesis and lipid peroxidation play essential roles in cell ferroptosis. Thus, we tested whether ACOT7 regulated fatty acid synthesis and lipid peroxidation. The results showed that ACOT7 knockdown enhanced malondialdehyde (MDA) production, triglyceride abundance, and the synthesis of oleic acid and palmitoleic acid (Fig. 5A-C). By contrast, ACOT7 overexpression suppressed MDA production, triglyceride abundance, and the synthesis of oleic acid and palmitoleic acid (Fig. 5D-F). These results suggest that ACOT7 play important role in regulating monounsaturated fatty acid metabolism.

### ACOT7 acts as a pivotal contributor for the proliferation of NSCLC cells with ARNTL2 overexpression

**Fig. 6:**
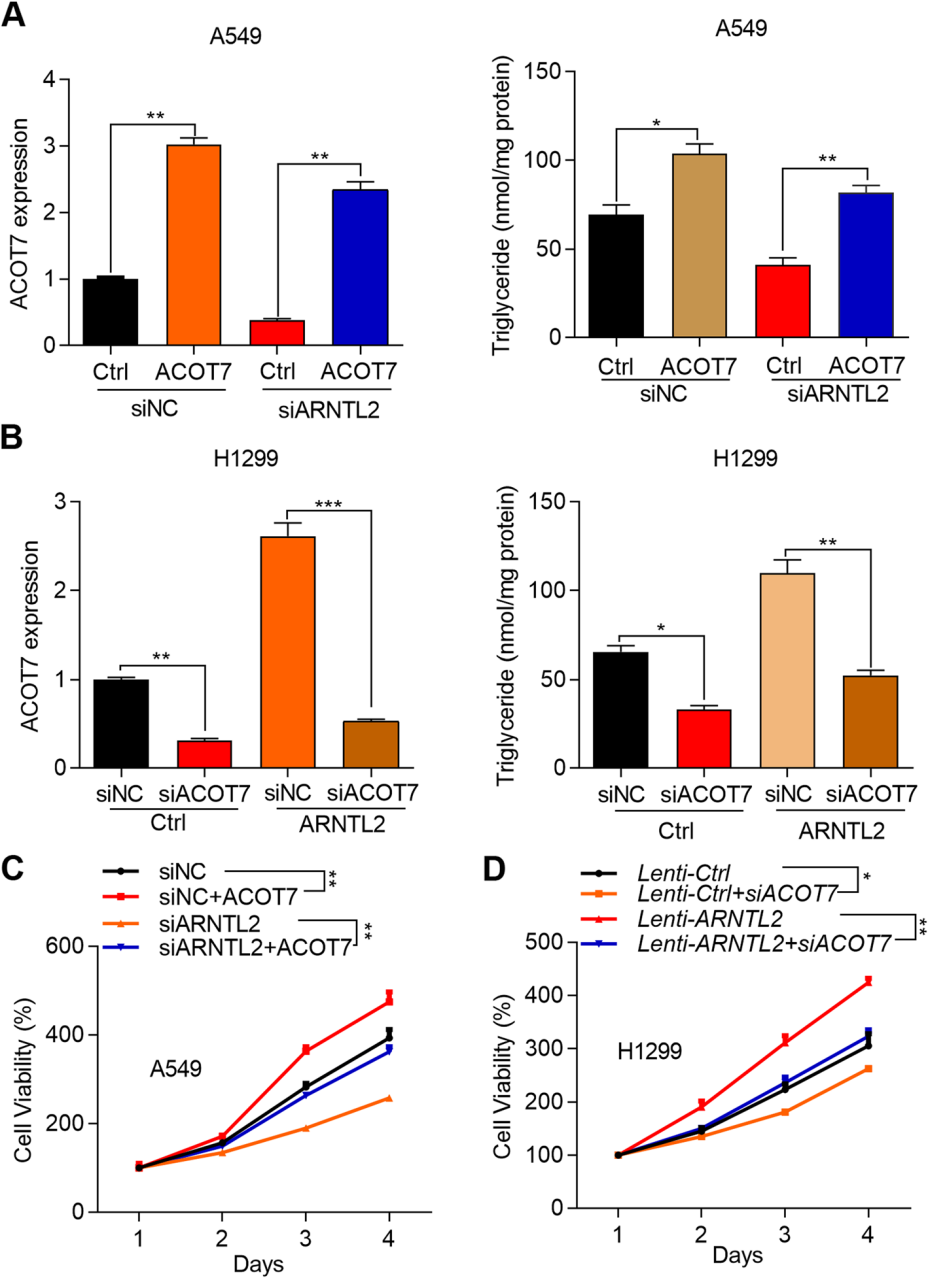
ACOT7 acts as a pivotal contributor for the proliferation of NSCLC cells with ARNTL2 overexpression. **A** A549 cells transfected with siNC, siNC + ACOT7 lentivirus, siARNTL2 + Ctrl lentivirus and siARNTL2 + ACOT7 lentivirus subjected to RT-qPCR analysis of ACOT7 and detection of triglyceride. *p < 0.05. **p < 0.01. **B** H1299 cells transfected with Ctrl lentivirus, Ctrl lentivirus + siACOT7, ARNTL2 lentivirus + siNC and ARNTL2 lentivirus + siACOT7 were subjected to RT-qPCR analysis of ACOT7 and detection of triglyceride. *p < 0.05. **p < 0.01. ***p < 0.001. **C** A549 cells described in A were subjected to CCK-8 analysis of cell proliferation. **p < 0.01. **D** H1299 cells described in B were subjected to CCK-8 analysis of cell proliferation. *p < 0.05. **p < 0.01

Lastly, we tested whether ACOT7 upregulation contributed the oncogenic function of ARNTL2 in NSCLC cells. To address this question, we overexpressed ACOT7 in A549 cells transfected with siNC or siARNTL2 and knocked down ACOT7 in H1299 cells transfected with *Lenti-Ctrl* or *Lenti-ARNTL2*. Firstly, we observed that ACOT7 overexpression promoted, while ACOT7 knockdown suppressed the triglyceride production and proliferation ability of NSCLC cells transfected with control lentivirus or siRNAs (Fig. 6AD). In addition, we showed that ACOT7 overexpression also increased the production of triglyceride in ARNTL2 knockdown cells (Fig. 6A). Opposite results were observed in H1299 cells with ARNTL2 overexpression and ACOT7 knockdown (Fig. 6B). Importantly, ACOT7 overexpression rescued the cell proliferation of A549 cells which was suppressed by ARNTL2 knockdown (Fig. 6C). On the contrary, ACOT7 knockdown suppressed the proliferation of H1299 cells which was promoted by ARNTL2 overexpression (Fig. 6D). In summary, ARNTL2 promotes NSCLC cell proliferation through upregulation of ACOT7.

## Discussion

During the past decades, a wide range of studies have shown that dysregulations of circadian rhythms genes, including Clock, Period 1, Period 2 and Period 3, and Bmal1, play essential role in the development of various cancers [13]. Comparing to these proteins, the involvement and molecular mechanisms of another circadian rhythms gene, named Bmal2 (ARNTL2), was less clear in the development of malignant tumors. Based on recent studies, the significance of ARNTL2 in carcinogenesis has been addressed. ARNTL2 upregulation promotes pancreatic ductal adenocarcinoma development via potentiation of TGF-β signaling pathway [9]. ARNTL2 is identified as a susceptibility gene for the metastasis of breast cancer with estrogen receptor-negative characteristic [10]. In addition, overexpression of ARNTL2 contributes to the metastatic ability of lung cancer through regulating pro-metastatic secretome [11]. These studies suggest that ARNTL2 exhibits important functions during carcinogenesis and metastasis. In this study, we illustrated that ARNTL2 was overexpressed in LUAD and LUSC patients. Overexpression of ARNTL2 conferred poor prognosis of LUAD, but not LUSC patients. Based on gain-of-function and loss-of-function experiments, we demonstrated that ARNTL2 promoted NSCLC cell proliferation and growth. Consistent with previous studies, our study showed that ARNTL2 overexpression was critical to maintain the function of NSCLC cells.

Since ARNTL2 is a transcription factor, the downstream effectors which are essential for the oncogenic function of ARNTL2 should be investigated. Increasing evidence has reported that dysregulation of ARNTL2 is involved in regulating the metabolism of fatty acid [12, 14, 15]. Nevertheless, whether ARNTL2 regulates the expression of fatty acid-associated genes should be determined. The acyl-CoA thioesterase (ACOT) genes encode the enzymes that catalyze the production of free fatty acids and CoA derived from fatty acyl-CoA [16]. Abnormal expression of different ACOT proteins has been shown to participate in the development of cancers. For example, ACOT4 expression is potentiated in pancreatic ductal carcinoma (PDAC). Depletion of ACOT4 blunts PDAC tumorigenesis [17]. In clear cell renal cell carcinoma (ccRCC), the majority of ACOT members, including ACOT1, ACOT2, ACOT3, ACOT8 and ACOT11, are downregulated in the cancer samples [18]. On the contrary, the role of ACOT11 in lung cancer is different from its role in ccRCC. ACOT11 is upregulated in lung adenocarcinoma. Knockdown of ACOT11 alleviates the proliferation, migration and tumorigenesis of lung cancer cells [19]. These results suggest that the role of ACOT proteins is distinct among various cancer types. Comparing with other ACOTs, ACOT7 is more frequently reported as cancer promoter by various studies. ACOT7 promotes cell cycle progression by regulating p53-p21 signaling pathway in breast cancer [20]. In acute myeloid leukemia patients, higher expression of ACOT7 predicts the poorer prognosis of the patients [21]. Furthermore, ACOT7 is positively regulated by transcription factor krüppel-like factor 13 (KLF13). Activation of KLF13/ACOT7 axis supports the progression of hepatocellular carcinoma (HCC) through potentiation of oleic acid [22]. These results illustrate that ACOT7 exhibits oncogenic potential in different cancers. However, the upstream controller and the significance of ACOT7 in NSCLC are less clear. Here, we demonstrated that ACOT7 expression was stimulated by circadian rhythms transcription factor ARNTL2. ARNTL2 and ACOT7 expression showed positive spearman correlation and their high expression was worse predictor for LUAD patients. Importantly, ACOT7 expression was critical to maintain the proliferation ability of NSCLC cells. The oncogenic function of ARNTL2 could also be reversed by changing the expression of ACOT7. Therefore, ACOT7 serves as an oncogene in NSCLC patients. ARNTL2 promotes NSCLC progression through positive regulation of ACOT7.

Ferroptosis is a specific type of cell death which is closely correlated with fatty acid metabolism, such as lipid peroxidation [23]. Suppression of ferroptosis plays a pivotal role in the contribution of cancer development [24, 25]. Unlike apoptosis, which is positively stained by Annexin V, ferroptosis is specifically stained by PI [25]. Initially, we observed that ACOT7 downregulation promoted, while ACOT7 overexpressed suppressed the percentage of Annexin V positive cells. The activity of caspase 3 to caspase 7 was repressed by ACOT7, indicating that ACOT7 inhibits the apoptosis of NSCLC cells. Additionally, ACOT7 knockdown also enhanced the percentage of PI positive cells, revealing that ACOT7 could regulate other types of cell death besides apoptosis. Various studies reported that lipid ROS was another hallmark of ferroptosis [24, 26]. By staining the cells with C-11 BODIPY, we found that ACOT7 negatively regulated the lipid ROS. To further validate the role of ACOT7 on ferroptosis, NSCLC cells transfected with different siRNAs were treated with ferroptosis or apoptosis inhibitors, the cell death triggered by ACOT7 knockdown could be reversed by the inhibitors alone or their combination. Furthermore, ACOT7 overexpression dramatically rescued the cell death triggered by ferroptosis, apoptosis inducer, or their combination. These results demonstrate that ACOT7 suppresses apoptosis and ferroptosis in NSCLC cells. Consistent with its role in HCC cells [22], ACOT7 positively regulated the production of oleic acid, as well as the cellular abundance of oleic acid, palmitoleic acid and triglyceride, suggesting that ACOT7 plays an important role in the synthesis of monounsaturated fatty acids. However, whether ACOT7 modulates the production of polyunsaturated fatty acids (PUFAs), such as arachidonic acid, should be explored in the future. Moreover, ACOT7 suppressed lipid peroxidation, as shown by reduced MDA level in ACOT7 overexpressed cells and by enhanced MDA level in ACOT7 knockdown cells. These results reveal that ACOT7 has important role in regulating fatty acid synthesis and lipid peroxidation. We also observed that ARNTL2 promoted the production of triglyceride, which could be reversed by changing the expression of ACOT7. Therefore, ARNTL2 upregulation of ACOT7 suppresses NSCLC cell apoptosis and ferroptosis by regulating fatty acid synthesis and lipid peroxidation.

## Conclusion

In summary, we identified that ARNTL2 transcriptionally upregulation of ACOT7 suppressed the apoptosis and ferroptosis, resulting in their promting function on the growth and proliferation of NSCLC cells. Mechanistically, ACOT7 modulation of fatty acid synthesis and lipid peroxidation could explain its dual role in apoptosis and ferroptosis. Our study provided an important clue that ARNTL2/ACOT7 contributed to NSCLC progression and could be targeted for the treatment of this deadly malignancy.

## Methods

### Analyzing ARNTL2 and ACOT7 in LUAD from TCGA

Gene Expression Profiling Interactive Analysis (GEPIA: http://gepia.cancer-pku.cn/) is a public database which analyzes the data from TCGA. We analyzed the expression of ARNTL2 and ACOT7, correlation between ARNTL2/ACOT7’s expression and patients’ survival, and the spearman correlation between ARNTL2 and ACOT7 in LUAD or LUSC patients based on TCGA database.

### Cell culture

Human immortalized normal lung cells Beas-2B and NSCLC cells A549, H1299 and H1975 were purchased from ATCC and were grew in RPMI-1640 complete medium, in which 10% fetal calf serum and 1% antibiotics were added. The cells were maintained in a 37 °C cell incubator in which the CO_2_ concentration was kept at 5%.

### RNA extraction and real-time quantitative polymerase chain reaction (RT-qPCR)

RNA was extracted from whole cell lysates using Trizol regent, following the manufacturers’ protocols. The RNA was reversely transcribed into cDNA using M-MLV reverse transcriptase (Promega). Quantification of the cDNA of indicated genes was conducted using SYBR master mixture (Takara) on Bio-rad real-time PCR system. The sequences of the primers were listed below: ARNTL2 forward, 5’-ACTTGGTGCTGGTAGTATTGGA-3’, and reverse, 5’-TGTTGGACTCGAATCATCAAGG-3’. ACOT7 forward, 5’-GGCCGGAAGCGGTATGAAG-3’, and reverse, 5’-GACTGGCTGTAGCTGACAGTG-3’. β-actin forward, 5’-GAGCTGCGTGTGGCTCCC-3’, and reverse, 5’-CCAGAGGCGTACAGGGATAGCA-3’.

### Immunoblotting

To assess the protein abundance in cells, we collected whole cell lysates from indicated cells using RIPA lysis buffer (Beyotime). Protein concentration was determined by BCA assay kit (Beyotime). 30–50 μg of the total proteins were separated on SDS-PAGE gels and immunoblotted onto PVDF membranes, which were activated by methanol. To avoid non-specific protein signal, the membranes were incubated with 5% skim milk dissolved in 0.1% PBST (99.9% PBS and 1% TWEEN-20). After incubating with secondary antibodies at room temperature for 2 h, protein signals were detected using chemiluminescence assay kit, according to the manufacturers’ protocols. Primary antibody against ARNTL2 was from Sigma-Aldrich (SAB2100154). ACOT7 (15,972–1-AP) and GAPDH (60,004–1-Ig) antibodies were from Proteintech. Anti-mouse (sc-2005) and rabbit (sc-2004) secondary antibodies were from SantaCruz.

### Cell proliferation and cell viability

Cell proliferation was analyzed by using CCK-8 assay kit (Beyotime). A549 cells transfected with siNC, siACOT7 (siACOT7#1 and siACOT7#2) or siARNTL2 (siARNTL2#1 and siARNTL2#2), and H1299 cells transfected with *lenti-Ctrl*, *lenti-ACOT7* or *lenti-ARNTL2*, were seeded in 96-well plates at a density of 3000 cells per well. Each well contained 100 μl RPMI-1640 complete medium. For the indicated time, 10 μl of CCK-8 buffer was added in each well and the plates were maintained in the cell incubator for 3 h. After vibrating the plates for 10 min, OD450 was measured on a microplate reader. The siRNAs were obtained from Hippo Biotechnology (Huzhou, China) and the sequences were as following: siNC: 5’-UUCUCCGAACGUGUCACGU-3’; siARNTL2#1: 5’-GAUGGUGAACACCAAGUUA-3’; siARNTL2#2, 5’-GGACAAGACCAACAGCUAU-3’; siACOT7#1, 5’-GCAUGACCUUCACGAGCAA-3’; siACOT7#2, 5’-CGCUGAAGAAUGUGGACAA-3’.

For cell viability analysis, indicated cells were incubated with DMSO, ferrostatin-1 (ferr-1, 2 μM, Selleck), Z-VAD-FMK (Z-VAD, 8 μg/ml, Selleck), erastin (5 μM, Selleck), apoptosis activator 2 (AA2, 4 μM, Selleck) for 6–10 h and cell viability was tested by trypan blue staining. Cell death (%) = 100%—cell viability (%).

### Colony formation

A total of 1000 siNC, siACOT7#1 and siACOT7#2 A549 cells and a total of 2000 siNC, siARNTL2#1 and siARNLT2#2 A549 cells were seeded in 6-well plates. A total of 500 lenti-Ctrl, *lenti-ARNTL2* and *lenti-ACOT7* H1299 cells were seeded in 6-well plates. 10 days later, colonies were formed. The plates were washed by PBS and the colonies were fixed by methanol and stained by crystal violet.

### Cell cycle

A549 cells transfected with siNC and siACOT7 (siACOT7#1 and siACOT7#2), and H1299 cells transfected with Ctrl and ACOT7 overexpression lentivirus, were seeded in 6-well plates. 48 h later, cells were harvested and maintained in 70% cold alcohol for 8–12 h. Subsequently, cell cycle was analyzed by PI staining (YEASEN, 40301ES50) on flow cytometry.

### Annexin V and PI staining

A549 cells transfected with siNC and siACOT7 (siACOT7#1 and siACOT7#2), and H1299 cells transfected with Ctrl and ACOT7 overexpression lentivirus, were seeded in 6-well plates. 48 h later, cells were harvested and subjected to PI/Annexin-V (YEASEN, 40302ES20) staining and analysis on flow cytometry.

### Lipid ROS detection on flow cytometry

Resuspended cells were stained with 3uM of C-11 BODIPY dye (Invitrogen) for 30 min, according to the manufacturers’ protocols. Fluorescence intensity was analyzed on flow cytometry.

### Caspase 3 to caspase 7 activity measurement

The activity of caspase 3 to caspase 7 was detected by using caspase-Glo reagent (Promega), according to the manufacturers’ protocols. In brief, after incubating the caspase-Glo reagent for 2 h, the activity of caspase 3 to caspase 7 was examined on a microplate reader.

### Dual luciferase reporter activity

Dual luciferase reporter activity was analyzed by using assay kit from Promega, according to the manufacturers’ protocols. The coding sequence of ARNTL2 was inserted into pCDNA3.1 vectors. The promoter sequence of ACOT7 (-2000 ~ 0 bp) was inserted into pGL3.basic vectors. Dual luciferase reporter activity was examined 48 h after co-transfecting siRNAs (siNC or siARNTL2)/pCDNA3.1 (pCDNA3.1-Ctrl or pCDNA3.1-ARNTL2), pGL3.basic vectors and TK vectors into NSCLC cells. Relative luciferase activity was adjusted to TK activity.

### Chromatin immunoprecipitation-qPCR assay

To assess whether ARNTL2 binds to the promoter sequence of ACOT7, we inserted the coding sequence of ARNTL2 into the pCDNA3.1-Flag plasmid. A549 and H1299 cells were transfected with the plasmids and subjected to chromatin immunoprecipitation assay by using SimpleChIP enzymatic chromatin IP kit (Cell Signaling), according to the manufacturer’s instructions. qPCR was applied to examine the DNA amplification samples in Chip-IgG and Chip-Flag group.

### Fatty acid metabolism

The cellular abundance of MDA in siNC, siACOT7#1 and siACOT7#2 A549 cells, and in lenti-Ctrl and lenti-ACOT7 H1299 cells was measured by using the assay kit from Beyotime (Shanghai, China), according to the manufacturer’s instructions. The cellular abundance of triglyceride in A549 and H1299 cells was assessed by using the assay kit from Nanjing Jiancheng, according to the manufacturer’s instructions.

### Statistical analysis

Statistical data were analyzed on GraphPad Prism 8 software and presented as mean ± standard error of mean (SEM). Students’t test was applied to determine the difference between two groups. One-way ANOVA followed by a Tukey’s post hoc test was used to determine the difference among groups. Statistical difference was considered significantly when *p* < 0.05.

## Supplementary Information

**Additional file 1: Supplementary Figure 1.** The clinical relevance of ARNTL2 in LUSC patients. **Supplementary Figure 2.** The expression of ARNTL2 in normal and cancer cell lines. **Supplementary Figure 3.** The effect of ACOT7 on the cell cycle of NSCLC cells. **Supplementary Figure 4.** The effect of ACOT7 on the activity of caspase 3 to caspase 7 in NSCLC cells. Original immunoblotting results.

## Data Availability

The data generated during this work were available in this article.
