# Supplementary material for: ARNTL2 upregulation of ACOT7 promotes NSCLC cell proliferation through inhibition of apoptosis and ferroptosis
Source: BMC Mol Cell Biol. 2023 Mar 31;24:14. doi: 10.1186/s12860-022-00450-5 (PMC10064581; doi:10.1186/s12860-022-00450-5)
Supplement: Supplementary file 1 — Additional file 1: Supplementary Figure 1. The clinical relevance of ARNTL2 in LUSC patients. Supplementary Figure 2. The expression of ARNTL2 in normal and cancer cell lines. Supplementary Figure 3. The effect of ACOT7 on the cell cycle of NSCLC cells. Supplementary Figure 4. The effect of ACOT7 on the activity of caspase 3 to caspase 7 in NSCLC cells. Original immunoblotting results. [file 12860_2022_450_MOESM1_ESM.docx]

**Supplementary Figure 1. The clinical relevance of ARNTL2 in LUSC patients.**

**
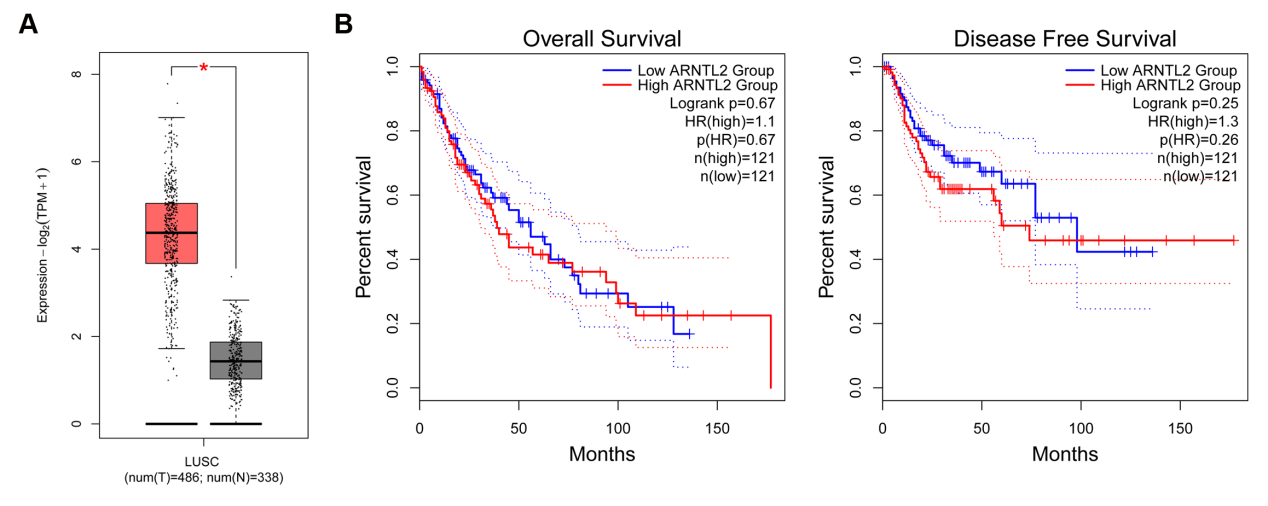
**

(A) The mRNA expression of ARNTL2 was analyzed in LUSC (n=486) and normal (n=338) tissues based on the TCGA database. p<0.05. (B) Overall and disease-free survival was analyzed for LUSC patients who were divided into ARNTL2 high and low expression group. n=121 per group. p>0.05.

**Supplementary Figure 2. The expression of ARNTL2 in normal and cancer cell lines.**

**
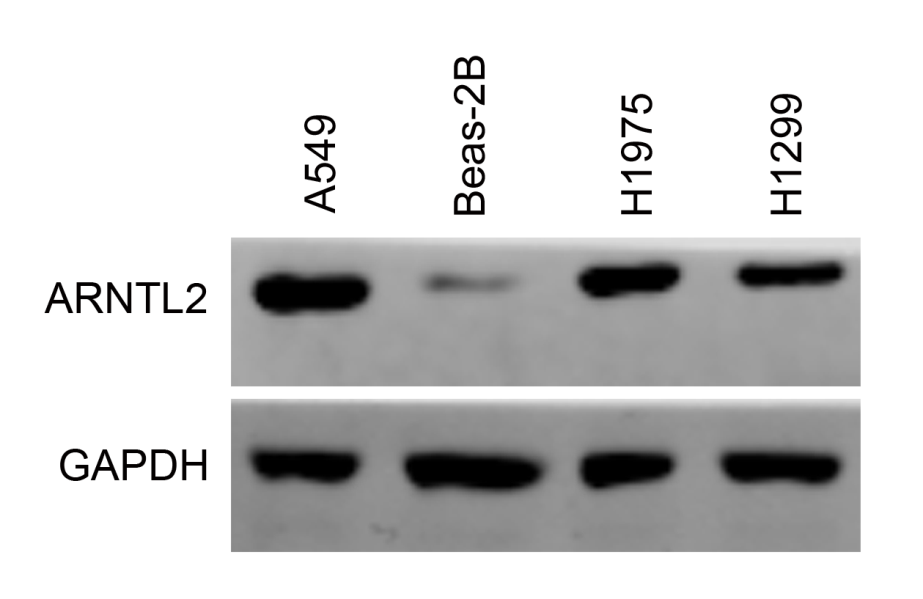
**

Immunoblotting analysis of ARNTL2 in normal lung cells Beas-2B and in NSCLC cells A549, H1299 and H1975.

**Supplementary Figure 3. The effect of ACOT7 on the cell cycle of NSCLC cells.**


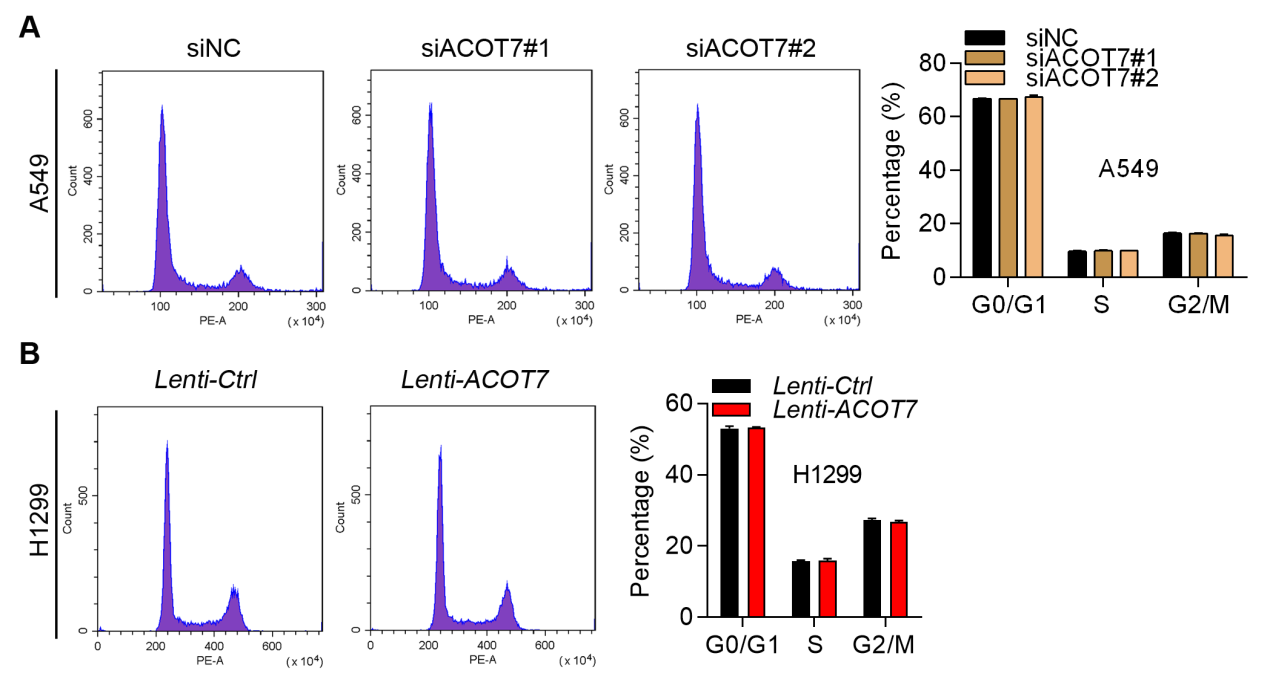


(A and B) A549 cells transfected with siNC, siACOT7#1 and siACOT7#2, and H1299 cells transfected with *Lenti-Ctrl* and in *Lenti-ACOT7* were subjected to PI staining and flow cytometry analysis of cell cycle. The difference was not statistically significant.

**Supplementary Figure 4. The effect of ACOT7 on the activity of caspase 3 to caspase 7 in NSCLC cells.**

**
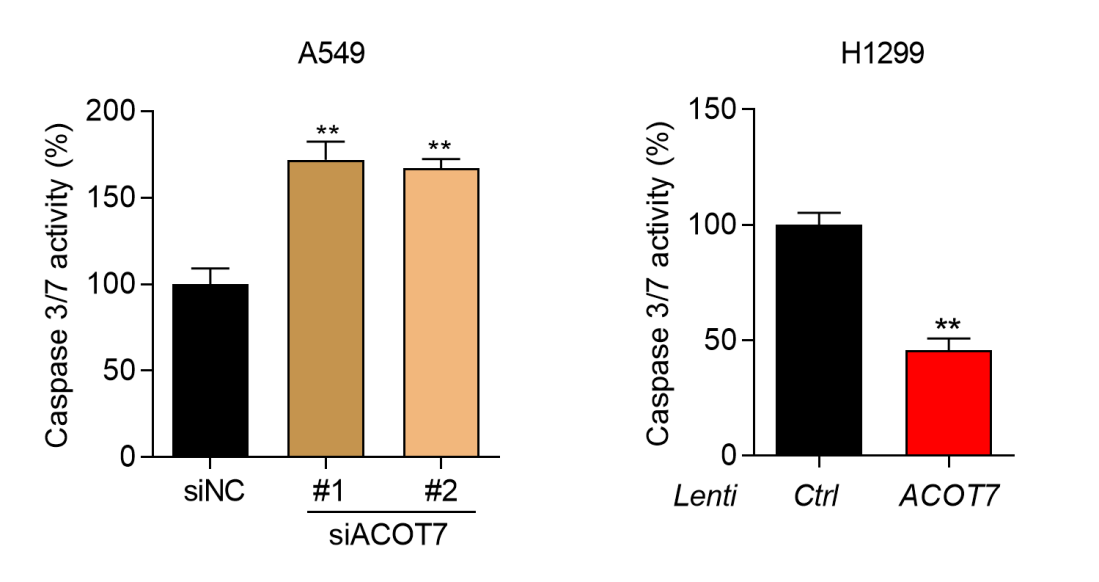
**

The activity of caspase 3 to 7 was examined in siNC, siACOT7#1 and siACOT7#2 A549 cells, and in *Lenti-Ctrl* and in *Lenti-ACOT7* H1299 cells*.* **p<0.01.

**Original immunoblotting results**

**
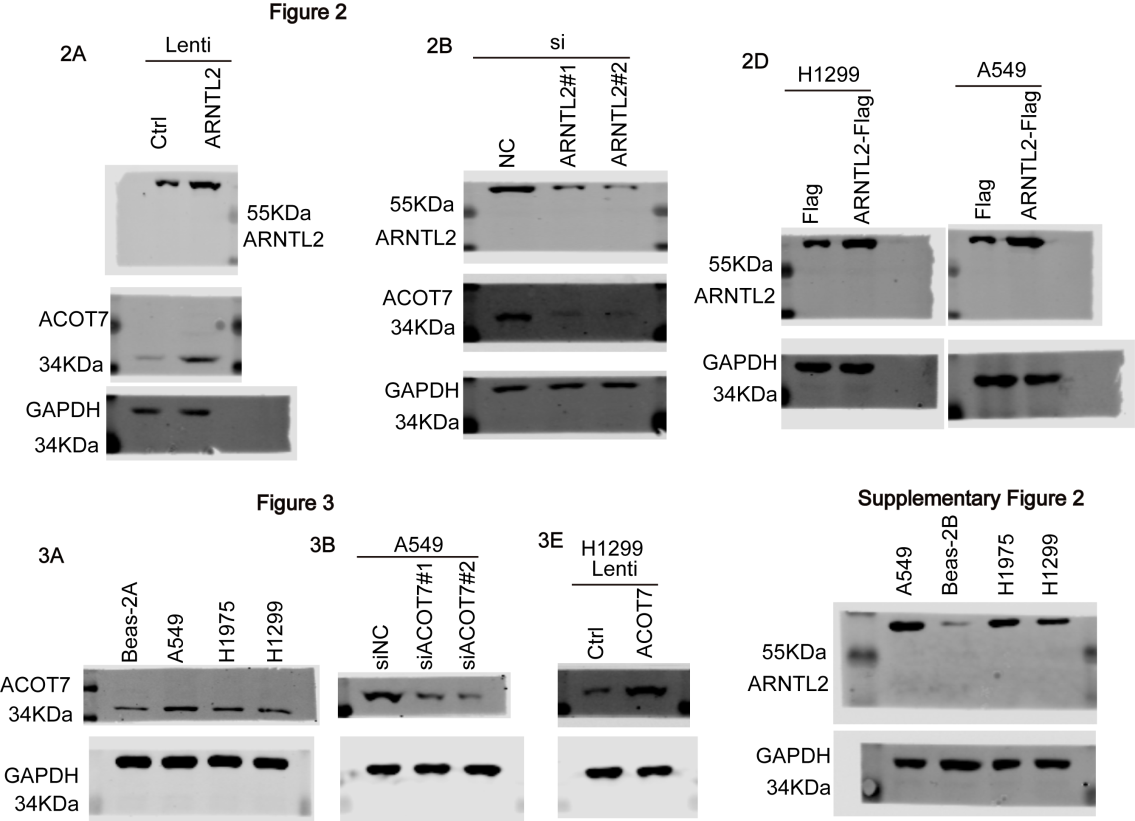
**
